# Potential neuroprotective and anticonvulsant effects of *Ganoderma lucidum* ethanol extract and its impact on affective comorbidities associated with pentylenetetrazol kindling model of epilepsy: behavioral, biochemical and histological studies

**DOI:** 10.3389/fneur.2026.1752481

**Published:** 2026-07-10

**Authors:** Itto Rahou Abdessamad, Bikjdaouene Leila, Bahbiti Youssef, Sqalli Houssaini Youssef, El Mekhlouf Youssef, Azeroil Fatima, Doumar Hanan, Dimaoui Amal, El-Hessni Aboubaker, Mesfioui Abdelhalem

**Affiliations:** 1 Laboratory of Biology and Health, Neurosciences, Neuroimmunology and Behaviour Unit, Faculty of Science, Ibn Tofail University, Kenitra, Morocco; 2 Higher Institute of Nursing and Health Technology of Tanger (ISPITS), Tangier, Morocco

**Keywords:** depression and anxiety, epilepsy, *Ganoderma lucidum*, oxidative stress, pentylenetetrazol

## Abstract

Epilepsy is a chronic neurological disorder that is strongly influenced by oxidative stress and neuronal death, two major pathophysiological factors. According to various recent studies, *Ganoderma lucidum* extract (GLE) may have antioxidant properties. However, there is little evidence to support its effectiveness in treating epilepsy. Here, we examined the neuroprotective qualities of GLE (300 mg/kg) against seizures induced by pentylenetetrazol (35 mg/kg). Four groups of eight male Wistar rats per group were randomly assigned: control, Pentylenetetrazol (PTZ), GLE-300 and Diazepam. The animals received an intraperitoneal dose of PTZ (35 mg/kg) every 48 h (11 injections in total) for 21 days to create an epilepsy model. The Racine five-point score was used to assess seizure severity. Anxious behavior, locomotor and exploratory activity were assessed using the open field test (OFT) and the elevated plus maze test (EPM), while the forced swim test (FST) was used to assess depressive behavior. Markers of oxidative stress, including malondialdehyde (MDA), nitric oxide (NO), and the enzymatic activities of superoxide dismutase (SOD) and catalase, were measured using the 2-thiobarbituric acid method (TBA), Griess reagent, nitro blue tetrazolium (NBT) reduction, and hydrogen peroxide decomposition. We performed a histological study of the prefrontal cortex (PFC) to quantify neuronal loss in the cortical layers using Nissl staining. All in all, our results showed that GLE reduces the severity of convulsive seizures during the induction of epilepsy (*p* < 0.001) and alleviates anxious (*p* = 0.005) and depressive (*p* = 0.008) behaviors while reducing neuronal damage. As well as significantly reduce NO and MDA levels (*p* < 0.001), and restore catalase (*p* = 0.005) and SOD (*p* = 0.010) enzyme activities in biochemical evaluation. Histologically, the GLE reduces neurodegeneration in the PFC due to its antioxidant and neuroprotective properties (*p* = 0.003). In conclusion, the experimental results indicate that GLE significantly attenuates the behavioral, biochemical, and histological alterations induced by PTZ, thus supporting its neurotherapeutic potential in the PTZ-Kindling model of epilepsy.

## Introduction

Fifty million people worldwide suffer from epilepsy, a serious and complex brain disorder (1). This neurological disorder severely affects health and quality of life, and its pathogenesis has yet to be fully elucidated.

In addition, recurrent epileptic seizures are a characteristic feature of this neuropathology, which results from an imbalance between inhibitory and excitatory electrical signals that play a crucial role in triggering neuronal degeneration in epilepsy (2, 3). Seizures may present in a clinic as convulsive seizures with clonic and/or tonic components, abnormal sensory and autonomic, or cognitive functioning (4).

Epilepsy patients are also prone to comorbidities like dementia, despair, and anxiety (5), and as the literature points out, most people who have epilepsy also have at least one other ailment (6). Individuals with epilepsy have an eight-fold greater frequency of depression, anxiety, and dementia compared to the overall population (5). Moreover, the PFC was selected because of its key role in regulating cortical excitability and affective functions, as well as its sensitivity to alterations induced by the PTZ-kindling model.

Epilepsy is characterized by neuronal hyperexcitability closely associated with an imbalance in the brain’s oxidative status (7). The pathophysiology of this condition is strongly influenced by oxidative stress, as evidenced by elevated markers of lipid peroxidation such as MDA and excessive NO production, coupled with a reduction in antioxidant defense mechanisms, particularly SOD and catalase (8). This state of redox imbalance promotes neuronal damage, compromises membrane integrity, and contributes to the onset and spread of epileptic seizures (9).

The PTZ-kindling model has been frequently used to study postseizure disorders and evaluate the potential treatment of epilepsy-related affective and emotional disorders in humans (10). The PTZ-induced generalized epilepsy kindling model in rats reveals neuronal hyperexcitability, accompanied by elevated biomarkers of oxidative stress, such as MDA and NO (11). There were also changes in antioxidant enzyme activity, notably catalase and SOD (12, 13).

Given the ineffectiveness or side effects of antiepileptic drugs, interest in natural products with neuroprotective and antioxidant potential continues to grow. Previous animal studies have shown that Triterpenes, Heteropolysaccharides, and Phenolic Compounds (14), protect against seizures and improve emotional and learning capacities (15). For more than 2000 years, Chinese medicine has used *G. lucidum*, a member of the basidiomycetes group, as an edible medicinal fungus because of its ability to increase lifespan and vigor (16). These mushrooms are currently very popular in the formulation of biotherapeutic products and nutritional supplements thanks to their high nutraceutical content, such as trace elements, dietary fibers, terpenoids, polysaccharides, proteins, minerals and phenolic compounds (17).

*Ganoderma lucidum* is a promising mushroom for epilepsy research due to its rich content of bioactive compounds such as phenolic compounds, flavonoids, ergosterol, and in particular, triterpenoids, namely ganoderic acids, which can modulate neuronal hyperexcitability (18–20). Experimental data indicate that *G. lucidum* can reduce NO and MDA levels, thereby helping to restore the catalytic activity of SOD and catalase, which are central to the pathophysiology of epilepsy (21–24). Furthermore, ganoderic acid A has shown antiepileptic and neuroprotective effects in the PTZ model, involving calcium regulation and inhibition of pro-apoptotic pathways, supporting the multi-target approach of *G. lucidum* in the treatment of epilepsy (18, 19).

The pharmacological effects of GLE are varied, with previous studies focusing mainly on antioxidant, antitumor, hypoglycemic, anti-inflammatory, hypolipidemic and immune regulatory effects (25). According to several studies, GLE has a strong neuroprotective impact against neurological conditions (23).

GLE has highly significant neuroprotective effects by preventing neuronal death caused by oxidative stress through the regulation of proteins associated with apoptosis (23). In recent years, research has shown that pre-administration of *G. lucidum* spores in rats can protect the PFC from oxidative damage (26).

In our study, we evaluated the potential anticonvulsant and neuroprotective therapeutic effects of GLE on seizures induced during kindling and affective comorbidities, focusing on its ability to modulate neuronal excitability and suppress epilepsy-related neurotoxicity.

## Experimental procedures

### Animals and laboratory

Adult male Wistar rats at 9 weeks of age and weighing 230 ± 17 g (obtained from Ibn Tofail University’s Animal House Department of Biology in Kenitra, Morocco) were housed in Plexiglas cages (21*29*43 cm) beneath controlled circumstances; Humidity (50–60%), Temperature (24 °C) and maintained on light/dark 12/12 h and had access to necessities (water and food). To minimize circadian influences on seizure susceptibility, convulsion induction was performed between 9:00 a.m. and 13:00 p.m.

### Mushroom preparation

**Figure 1:**
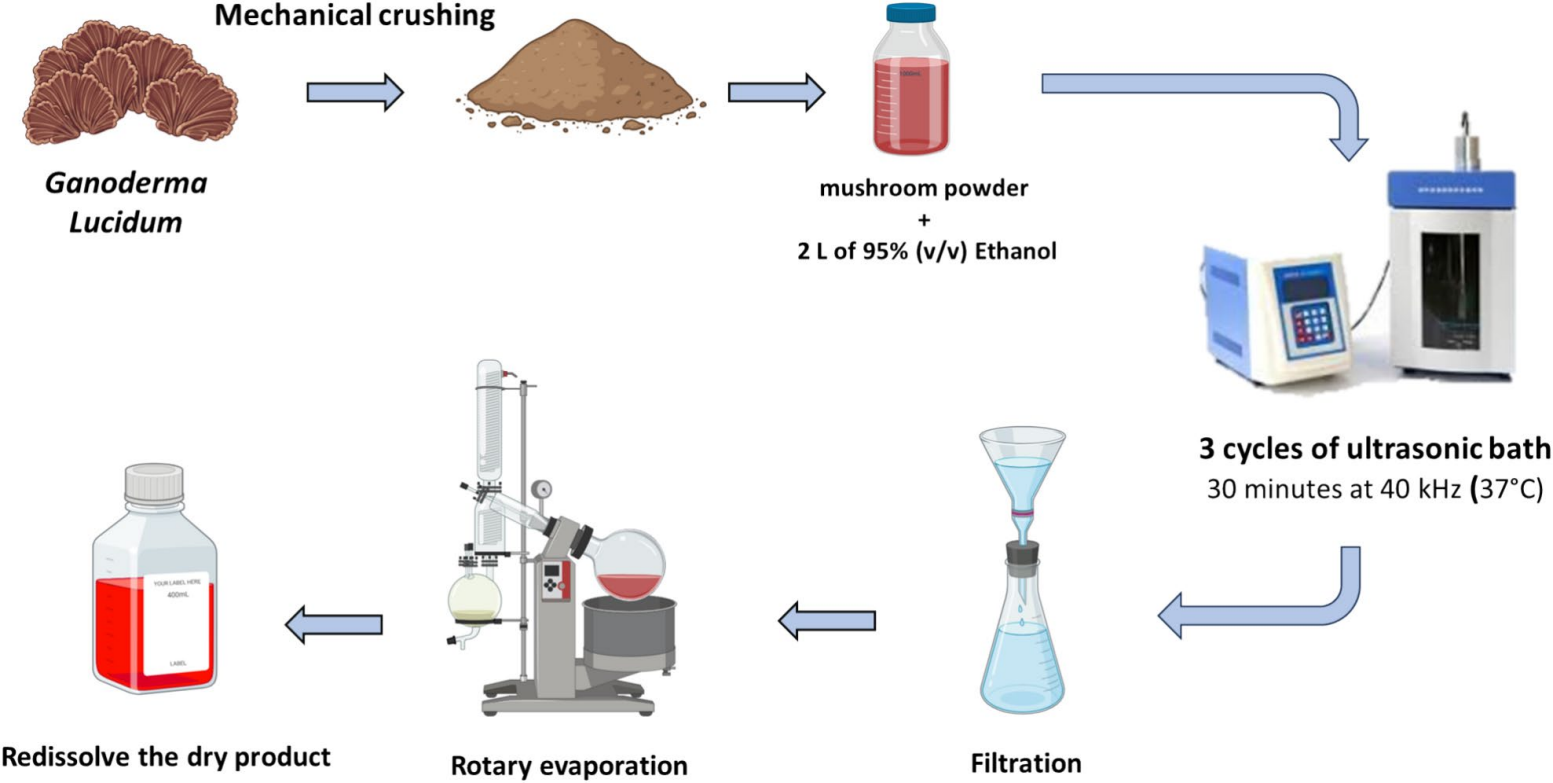
Schematic representation of the ultrasonic-assisted ethanol extraction process of *G. lucidum*.

In our study, the fruiting bodies of *G. lucidum* and the mycelium were ground to prepare the ethanolic extract of GLE. The powder of *G. lucidum* was mixed with ethanol, sonicated ultrasonically and dried, resulting in an extraction rate of 5.57%, which was redissolved in distilled water (DW) following the protocol in our prior study (27, 28) (see Figure 1).

### Chemicals and drugs

PTZ was obtained from Sigma Aldrich and *G. lucidum* mushrooms from DXN Pharmaceutical SDN. BHD (199601038339). The GLE was dissolved in DW and administered intragastrically (300 mg/kg; *i.g*) (29, 30). An intraperitoneal injection of (35 mg/kg; *i.p*) of PTZ dissolved in phosphate-buffered saline [PBS: 136.893 mM (NaCl), 2.683 mM (KCl), 10.144 mM (Na_2_HPO_4_), 1.8 mM (KH_2_PO_4_)] (Sigma Aldrich) was administered (31–33). Every treatment dosage utilized in the study was chosen based on previously published research. Diazepam (Sigma Aldrich D0899) was used for administration (Valium 10 mg/1 kg subcutaneous administration) (34, 35).

### Study design

4 groups of rats were randomly assigned (*n* = 8 for each group), the DW and GLE were administered by intragastric gavage. All the animals in other groups except the normal group had seizures, and none died during the experiment.

**Figure 2:**
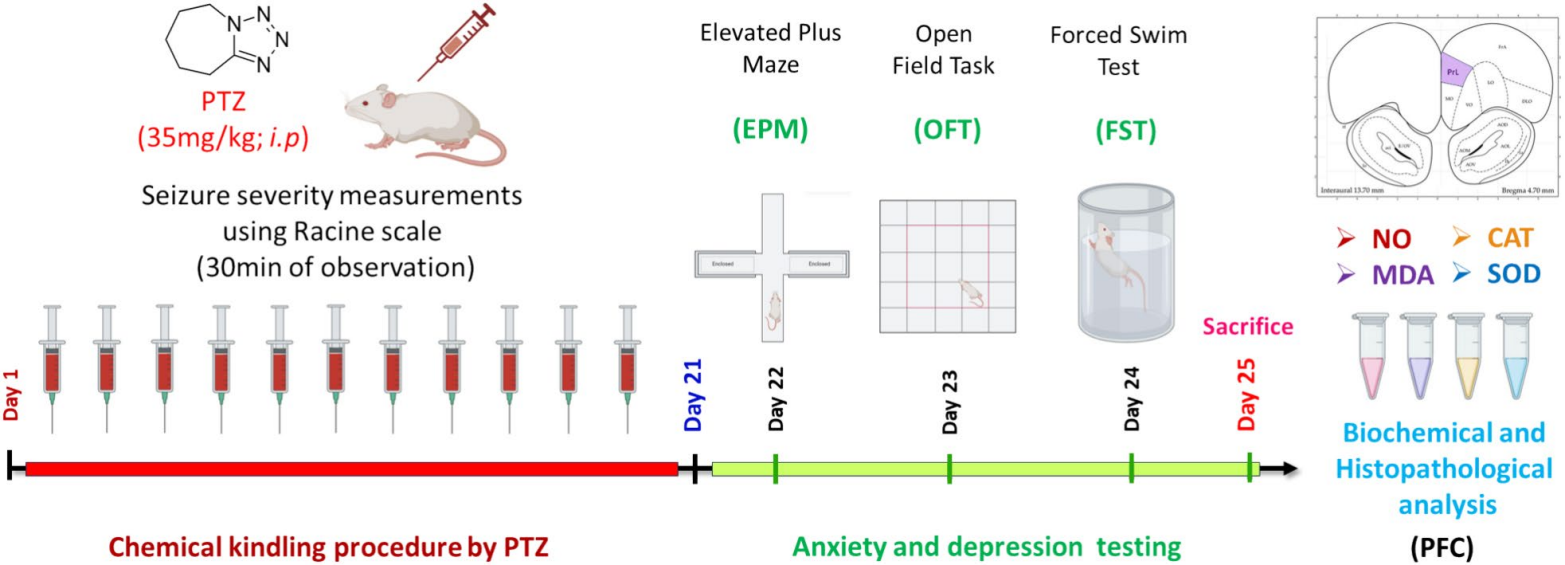
Experimental design of the PTZ-kindling model process and behavioral evaluation for depression and anxiety using the elevated plus maze test, forced swim, and open field tests. The day after the behavioral tests, the dissected brains underwent biochemical analyses to measure nitric oxide and malondialdehyde levels, as well as superoxide dismutase and catalase activity.

*Control group*: normal rats received 10 mL/kg of DW via intragastric gavage for 3 weeks (21 days) with 1 mL saline PBS via IP injection during the last 3 weeks, *PTZ group*: For 3 weeks (21 days), rats were intragastrically gavaged with 10 mL/kg of DW and then received a dose of PTZ (35 mg/kg in 1 mL of PBS, i.p) every 48 h for the last 2 weeks of the experiment. *GLE-300 group*: The animals were pretreated daily with GLE (300 mg/kg; i.g.) by intragastric gavage for 21 days (27, 29, 30), then received an intraperitoneal dose of PTZ (35 mg/kg; i.p.) every 48 h (11 injections in total) and *Diazepam group*: Diazepam (5 mg/kg, i.p.) was administered to the animals half an hour before PTZ administration (35 mg/kg in 1 mL of PBS; i.p.) (11 injections in total) every 48 h (36). In contrast, in the study of PTZ-induced kindling, diazepam is used as a predictive reference due to its effectiveness in preventing epileptic seizures. Figure 2 shows the study design. GLE dose selected based on previous studies (29, 30) and was administered intragastrically (i.g.) daily for 21 days (27, 37, 38).

### PTZ kindling procedure

To induce kindling, we administered a subconvulsive (35 mg/kg; i.p) dose of PTZ every 48 h, totaling 11 injections. Following each injection, rats were kept in clear Plexiglas cages and observed for half an hour, and the seizure behavior was evaluated in accordance with the Racine scale (Table 1) (33). After administering PTZ, the animals were considered fully kindled following three successive stage 4–5 seizures (39). Animals in the treatment groups were administered PTZ following 21 days of GLE pretreatment and half an hour after diazepam pretreatment. 24 h post the final PTZ injection, the rats underwent a series of behavioral assessments to evaluate affective performance (depression and anxiety), as illustrated in Figure 1. To avert any modification in animal behavior, each group received treatment at least 24 h before the behavioral testing. The behavioral tests were arranged for each research group in order of least to most stressful. The experimental apparatus was cleaned with diluted ethanol between each test to remove feces, urine, and odors, thereby preventing any influence on the animals’ behavior during subsequent tests. The rat cages were positioned in a behavioral room at least an hour before the experiment began to allow for adaptation, and the entire study was carried out from 9 h to 13 h. Following the behavioral examination, forty rats were sacrificed, and their entire brains were meticulously extracted for neurochemical analysis. The rats were sacrificed from 9:30 a.m. to 6:30 p.m.

**Table 1:** Racine scale.

Racine score	Behavioral characteristics
0	No behavioral changes
1	Facial movements, ear and whisker twitching
2	Unilateral forelimb convulsions
3	Bilateral forelimb complete convulsions
4	Clonic convulsion
5	Generalized clonic–tonic seizures

### Assessment of neurobehavioral parameters

#### EPM test

On the 42nd day, the animal’s anxiety and stress levels were evaluated using an EPM. This unit has a center platform (100*100 mm) that is 50 cm above the ground, as well as 2 open arms (500*100 mm) and 2 closed arms (500*100*500 mm). Initially, every rat was positioned separately in the middle, its head turned toward the outstretched arm. For 5 min, they were free to go about the maze. The number of entries in open arms (EOA) and the time spent in open arms (TOA) are two variables used to assess the level of anxiety.

Evaluation of the anti-anxiety effects of various pharmacological agents in rats is often carried out using the more complex maze test. While untreated rats generally refused to enter or remain in the open arms of the EPM, choosing the closed arms, rats that received the anxiolytics were less afraid of the open arms (40).

#### Open field test

OFT examines the impact of a pharmaceutical agent on anxiety behavior, locomotion and exploration behaviors in rodents. To achieve this, an OFT (800*800*450 mm) was used. On the 43rd day of the research, following the start-up process, Wistar rats were positioned one by one in the middle of the maze and observed for 10 min. Each animal underwent a single test, and once the test was completed for one rat, to eliminate any remnants of the previous animal’s scent, to clean the labyrinth, 70% isopropyl alcohol was used (41, 42). The test recordings were recorded and examined. Parameters such as the number of returns to the center (NRC) and time spent in the central area (TCA) were evaluated to assess the animal’s anxiety and motor activity, respectively (43).

#### Depression-like measurement

On the 44th day of the study, the rats were kept in separate glass tubes (d = 300 mm; h 500 mm) with 300 mm of water at a temperature of around (23 °C ± 2 °C) during swimming sessions. The rats were made to remain immobile for 5 min during the session, and the length of time was recorded. The immobility time was also noted, starting as soon as the rats were placed within the cylinder. When a rat ceased all active movement, such as swimming, jumping, and struggling and continued to passively float or make the necessary motions to maintain its nose above water, it was deemed immobile. A significant proportion of immobility time is estimated to increase depressive responses (44). Behavioral analysis was deliberately limited to immobility time, considered to be the most representative parameter of depressive-type behaviors. Swimming and climbing behaviors were not taken into account, as they were not part of the specific objectives of this study. The animals were monitored continuously and removed from the water in cases of distress or risk of drowning, then placed in a warm, calm environment until they recovered, in accordance with ethical recommendations for animal welfare. The temperature was checked before and between each test, with water added or replaced at the appropriate temperature to ensure consistent experimental conditions for all animals.

### Assessment of neurochemical parameters

#### Biochemical assessment

After testing for affective comorbidities (EPM, OFT, and FST), surviving rats were euthanized by cervical dislocation, and their brain structures were quickly removed and refrigerated on dry ice to measure oxidative damage. The PFC is dissected on ice. After being dissected individually, cold PFC are homogenized in 50 mM phosphate buffer (PB) (pH 7.4) using a Dounce homogenizer (Sigma-Aldrich). To measure the activity of catalase, NO, MDA, and SOD, the homogenate solutions were centrifuged at 3000 g for 30 min at 4 °C.

#### Catalase activity assay

Catalase activity was assessed using the Aebi method (45). 50 μL of supernatant was mixed with 1.95 mL PB [50 mM; pH = 7.5] in a cuvette. The addition of 1,000 μL of freshly prepared H2O2 (50 mM) triggered the catalytic reaction. Spectrophotometry has been used to measure the rate of H2O2 breakdown based on absorbance variations at a 240 nm spectrum at 25 °C. The enzymatic activity of catalase has been expressed in units international U/g, which is equivalent to μmol/min/g of Brain tissue.

#### Nitrite/nitrate assay

Quantification of nitrite, considered an indirect but reliable indicator of NO production, was carried out using the Griess colorimetric method (46). In brief, 100 μL of Griess reagent [0.1% N-(1-naphthyl) ethylenediamine hydrochloride; 1% sulfanilamide in 5% phosphoric acid; 1:1] was incubated with 100 μL of each sample at ambient temperature for 20 min. The absorbance was determined at 550 nm and then compared to that of standard sodium nitrite solutions. Concentrations obtained were normalized and expressed in μmol/g brain tissue, following the protocol of our previous study (27).

#### Lipid peroxidation assay

MDA is an effective indicator of oxidative stress. In our study, we examined the production of lipid peroxides by studying the reactions of thio barbituric acid in cells, according to Draper and Hadley’s description (1990). First, 1,000 μL of homogenates (PFC) were combined with [1,000 μL of Tri-Chloro-Acetic acid (10%) + 1,000 μL of Thio-Barbituric Acid (TBA; 0.67%)], 15 min of heating in boiling water (100 °C), for 15 min and the solution was then mixed with butanol (2:1 v/v). Following centrifugation at 8000 g/5 min, the MDA were identified by studying their absorbance at 535 nm (47–49).

#### SOD assay

The enzymatic activity of SOD was measured using the methodology described by Fridovich and Beauchamp (50). In the presence of oxygen and electron sources such as methionine, riboflavin illumination produces superoxide anions that prevent the reduction of nitro blue tetrazolium (NBT). The 1 mL reaction mixture consisted of 60 μL of supernatant and 940 μL of PB (50 mM) containing methionine (12 mM), NBT (75 μM), EDTA (0.1 mM), Triton X-100 (0.025%), and riboflavin (2 μM) at [pH 7.4]. By subjecting the mixture to 10 min of yellow light, SOD was measured. Additionally, an enzyme-free reference combination was added. Absorbance at 560 nm was used to quantify the quantity of NBT that superoxide radicals in blue formazan depleted. The quantity of enzyme needed to prevent a 50% drop in NBT under the previously stated circumstances is known as one unit of SOD activity (50, 51). This activity was expressed in U/g of brain tissue.

#### Nissl staining

To perform a histological evaluation of the PFC, four rats per group were used. They received a dose of 7% chloral hydrate (5 mL/kg; i.p.) to induce deep anesthesia. They were then given PBS solution intracardially for 2 min, followed by 4% paraformaldehyde in PBS (0.1 M; pH 7.4) for histological evaluation of the brain. After being removed, the brains were post-fixed in the same fixative for 12 h and cryopreserved for 24 h at 4\u00B0C in 20% sucrose/0.1 M PBS.

The next day, a vibratome (Leica VT1000 S, China) was used to take 30 μm serial coronal sections, which were then stored in glycerol solution at 4 °C. After being mounted on slides covered with gelatin, each portion was left to dry overnight. For histological analysis, staining was done using cresyl violet as previously mentioned (52). Coronal sections of the brain corresponding to the anterior PFC were analyzed, located between +4.94 mm and +3.20 mm anterior to bregma, with medio-lateral coordinates of ±0.11 to ±0.93 mm and dorsoventral depths ranging from −3.4 to −3.9 mm, according to the stereotaxic atlas of the rat brain. The light microscope (OPTIKA, Italy) equipped with a professional HDMI camera was used to observe the PFC neurons. For each animal, the observation areas were chosen systematically and comparably based on coronal sections corresponding to the same anatomical level of the prelimbic prefrontal cortex, as defined by the Paxinos and Watson atlas. Several distinct fields were examined bilaterally to minimize selection bias. The analysis focused specifically on cortical layers II/III and V of the prelimbic cortex. Neurons were considered intact when they had normal morphology, homogeneous cytoplasm, and a well-defined nucleus, while altered cells were identified by signs of nuclear pyknosis, cell shrinkage, cytoplasmic vacuolization, or loss of tissue integrity. To illustrate variations in the quantity and dimensions of morphological components, sections were examined at multiple magnifications (x10 and x40). Neuronal death was estimated indirectly by quantifying surviving neurons and comparing them with a control group.

### Statistical analysis

GraphPad Prism (version 8.0) was used for statistical analysis. The normality of distributions was verified using the Shapiro–Wilk test before applying parametric tests. Data are presented as mean ± SEM. Comparisons between groups for biochemical parameters, affective behaviors, biomarkers of oxidative stress, and the number of surviving cells in the PFC were performed using one-way ANOVA, followed by Tukey’s *post hoc* test when significant differences were detected. Racine scores were analyzed using a two-way ANOVA, followed by Tukey’s post hoc test for multiple comparisons. The GLE and diazepam groups were compared to the PTZ-kindled group (#) and with the healthy control group (*), in order to evaluate variations in biochemical markers. A *p*-value < 0.05 was considered statistically significant.

## Results

### Effect of GLE supplementation and diazepam treatment on the severity of convulsive seizures during the kindling process

**Figure 3:**
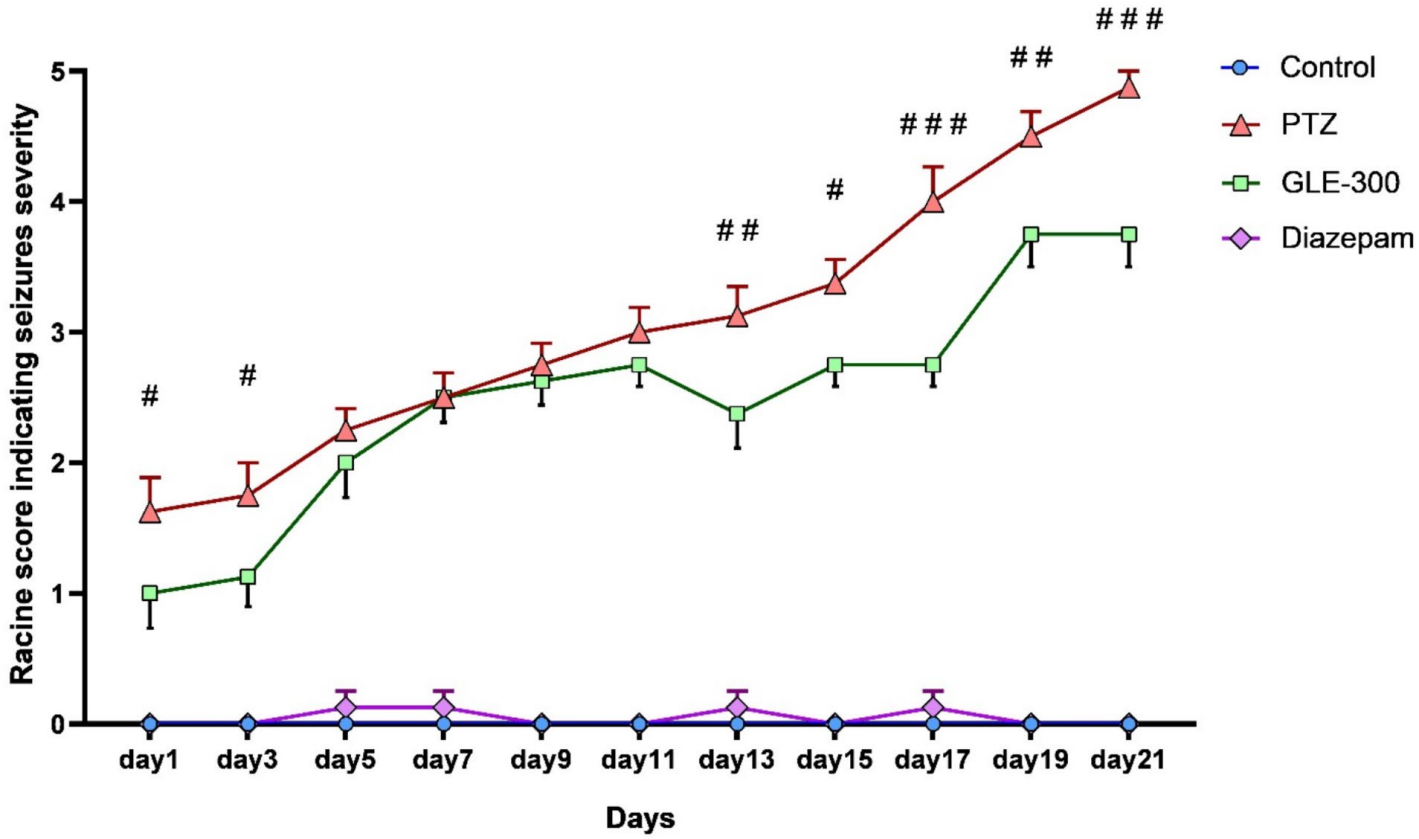
Effect of diazepam (5 mg/kg) and GLE (300 mg/kg) on the development of the PTZ-induced kindling process. The animals received PTZ injections every 48 h for 21 days at a dose of 35 mg/kg. Thirty minutes after each injection, the animals were observed to score the severity of their seizures using the Racine score. Two-way ANOVA and Tukey’s *post hoc* test were used for statistical analysis, and all data are reported as mean ± SEM (*n* = 8). For the GLE-300 group vs. the PTZ control (# *p* < 0.05, ## *p* < 0.01 and ### *p* < 0.001).

The statistical analysis revealed differences in seizure activity between the testing groups [*F* (30, 308) = 13.68; *p* < 0.001; Figure 3]. Animals in the PTZ group showed a significant increase in seizure severity, from an average stage of 1.625 ± 0.263 on day 1 to an average stage of 4.875 ± 0.125 on day 21, which corresponds to generalized tonic–clonic seizures. In contrast, the control group did not experience any seizures during the study (*p* < 0.001). Furthermore, the GLE-300 group showed a decrease in seizure intensity that was rather constant throughout the trial. With a maximum score of 3.75 ± 0.25, this group significantly outperformed the PTZ group (*p* < 0.001). The rats in the diazepam group had the lowest amount of kindling (0.125 ± 0.125 score) in comparison to the other kindled rats (*p* < 0.001), demonstrating how well the drug shielded the animals against the effects of PTZ. Consequently, our findings show that the administration of GLE reduces the degree of epileptic activity. Eventually, 87.5% of animals in the PTZ group had a score of 5 at day 21. However, during the test, none of the rats treated with diazepam scored 5. In the GLE-300 group, only one rat scored 5. Therefore, there was a statistically significant difference between rats in the diazepam and GLE-300 treatment groups (*p* < 0.001) at the end of the kindling phase. Overall, these results show that GLE supplementation significantly lessens the intensity of seizures.

### Effect of GLE pre-treatment on affective function

#### GLE’S impact on EPM’S anxious behavior

**Figure 4:**
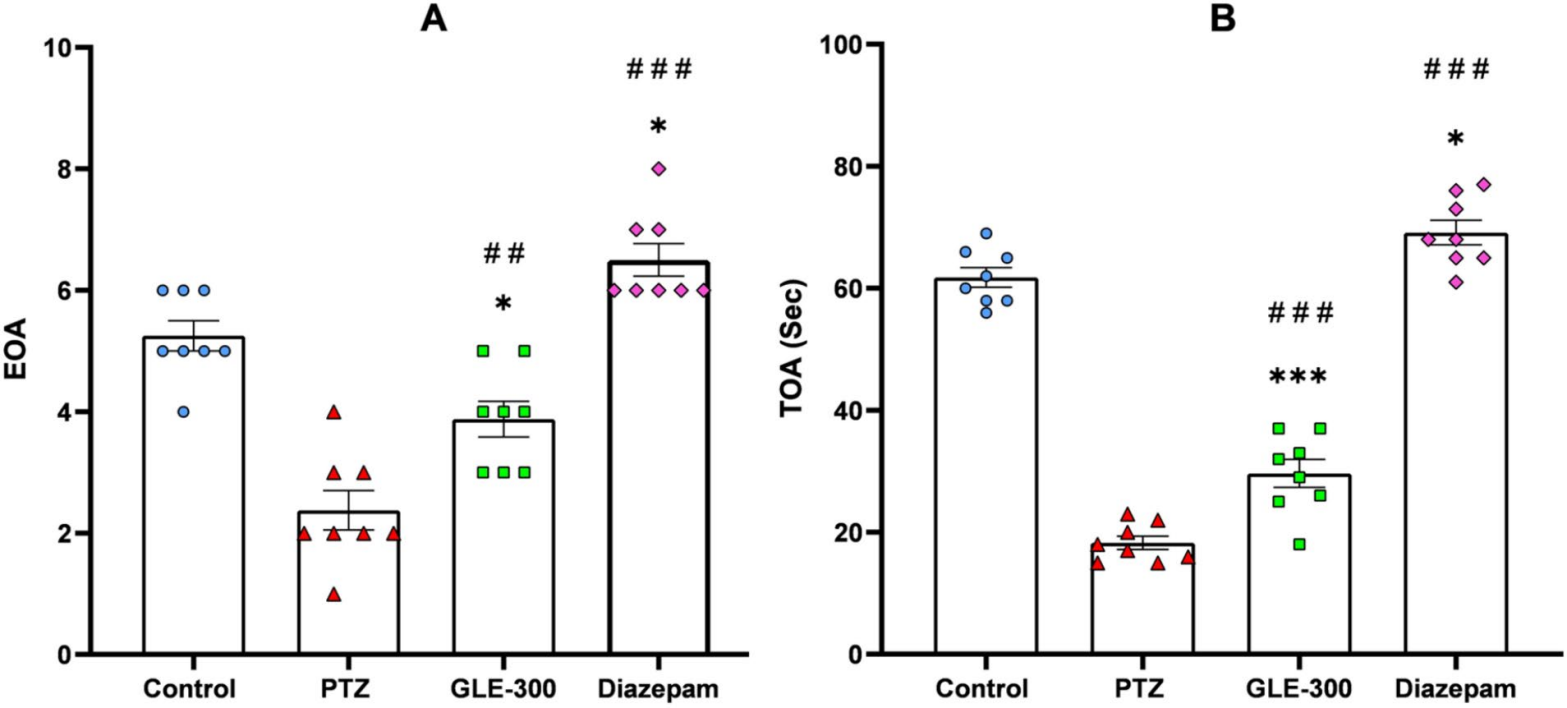
GLE’s effect on the EPM’s behavioral metrics. **(A)** Number of entries in open arm (EOA) and **(B)** Time spent in open arm (TOA) (sec). The results were presented as (mean ± SEM; *n* = 8). One-way ANOVA was performed using Tukey’s post hoc test. *p < 0.05 and ***p < 0.001 denote significant differences between the diazepam and GLE-300 groups and the control. Conversely, ##*p* < 0.01, and ###*p* < 0.001 indicate significant differences between the diazepam and GLE-300 groups and the PTZ group.

One-way ANOVA in EPM showed that EOA [*F* (3, 28) = 38.7397, *p* < 0.001; Figure 4A] and TOA [*F* (3, 28) = 184.106, *p* < 0.001; Figure 4B] varied significantly across all groups in the EPM test. Rats of the PTZ group showed a significant decrease in EOA (2.375 ± 0.3239 vs. 5.250 ± 0.2500; *p* < 0.001) and TOA (18.25 ± 1.098 Sec vs. 61.75 ± 1.612 Sec; *p* < 0.001) in the EPM test compared to control animals (*p* < 0.001), reflecting marked anxious behavior. Furthermore, in contrast with PTZ-kindled rats, GLE-300 and Diazepam administration increased the frequency of EOA by (3.875 ± 0.295; *p* = 0.005) and (6.500 ± 0.267; *p* < 0.001), respectively. However, the impact was more noticeable in the GLE-300 (29.63 ± 2.299; *p* < 0.001) and diazepam group (69.13 ± 2.013; *p* < 0.001) as animals resided in open arms longer as compared to the PTZ control. Overall, the post-hoc test showed that pre-treatment with GLE-300 and diazepam exerted a notable anxiolytic effect in comparison to kindled rats.

#### GLE’S impact on anxiety-like conduct during the open-field test

**Figure 5:**
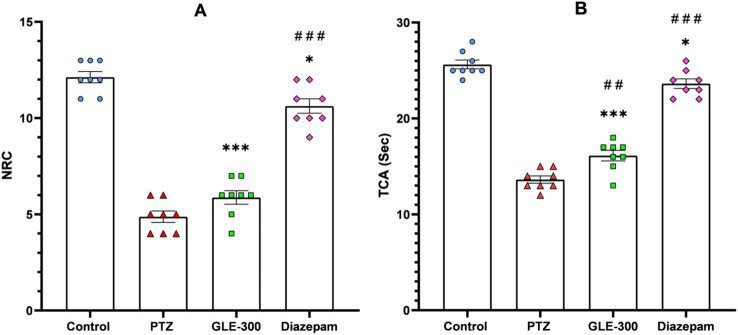
Post-kindling anxious behavior was measured using the open field test (OFT) to investigate how rats’ movement was affected by long-term administration of GLE (300 mg/kg; *i.g*). The animal’s activity was further evaluated for their **(A)** number of returns to the center (NRC) and **(B)** time spent in the central area (TCA) (Sec) to examine the anxiolytic effects of GLE. The results were compared with the PTZ control rat receiving diazepam as a conventional anxiolytic using a one-way ANOVA and Tucker’s post hoc test. Significant differences between the diazepam and GLE-300 groups and the control are indicated by *p < 0.05 and ***p < 0.001; significant differences between the diazepam and GLE-300 groups and the PTZ group are indicated by ##*p* < 0.01, and ###*p* < 0.001.

To assess the rats’ locomotor activity and anxiety-like behavior, they were given free rein to explore an open field labyrinth. All groups showed a statistically significant difference for NRC, according to the one-way ANOVA [*F* (3, 28) = 114.667, *p* < 0.001] and TCA [*F* (3, 28) = 148.132, *p* < 0.001] as shown in Figures 5A,B.

The healthy animals indicated lower levels of anxiety in the OFT and frequented the central area more regularly and their number of recorded entries was 12.13 ± 0.29, with a prolonged time spent in the central area of 25.63 ± 0.4605 Sec (Figures 4A,B). Conversely, the PTZ control rats showed signs of anxiety, as evidenced by a drop in their central area entries to 4.87 ± 0.29. Their shorter core zone exploration time of 13.63 ± 0.37 Sec provided more evidence for this. GLE reduced this anxiety related to kindling, and rats treated with GLE-300 showed significantly better results. In comparison to the PTZ control, the animals that received GLE-300 approached the middle region 5.87 ± 0.35 (*p* = 0.166) and remained there for 16.13 ± 0.54 Sec (*p* = 0.005). The animals receiving Diazepam entered the central area for 10.63 ± 0.37 (*p* < 0.001) and stayed there for 23.63 ± 0.49 Sec (*p* < 0.001), as compared to the PTZ control.

#### Rats exposed to PTZ-induced kindling in the FST: the impact of GLE

**Figure 6:**
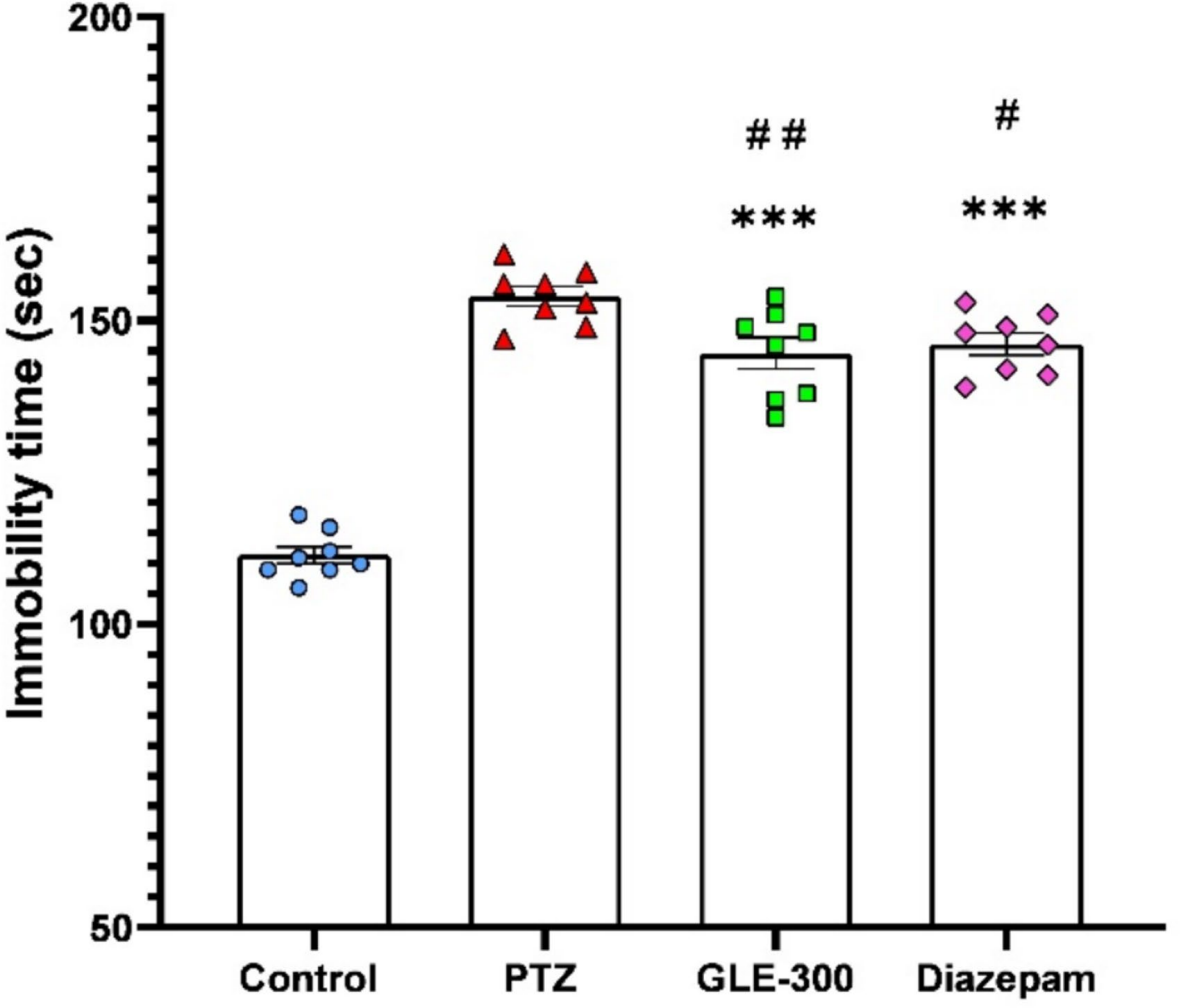
Recurring GLE pretreatment’s impact on the PTZ-kindled rats’ depressive-like behavior during the forced swim test. One-way ANOVA and Tukey’s post hoc test were used for the statistical analysis, and all data are shown as mean ± S.E.M (*n* = 8). ***p < 0.001 represent statistically significant differences within the diazepam and GLE-300 groups and the control group; furthermore, #*p* < 0.05 and ##*p* < 0.01 show statistically significant differences between the diazepam and GLE-300 groups and the PTZ group.

The FST was used to assess the rats’ depressive-like behavior. All groups’ immobility durations differed statistically significantly, according to the one-way ANOVA [*F* (3, 28) = 98.365, *p* < 0.001; Figure 6], as illustrated in Figure 5 in Tukey’s test. The PTZ group showed a significant increase in immobility time (*p* < 0.001) at 154.00 ± 1.648 Sec, compared to the control group at 111.37 ± 1.388 Sec. This indicates depressive behavior. However, GLE significantly reduced the duration of immobility in animals in the GLE-300 group 144.6 ± 2.591 Sec compared to the PTZ group (*p* = 0.008), while diazepam had little effect 146.1 ± 1.777 Sec (*p* = 0.032).

### Effects of repeated administration of GLE and diazepam on oxidative stress parameters

#### NO levels

**Figure 7:**
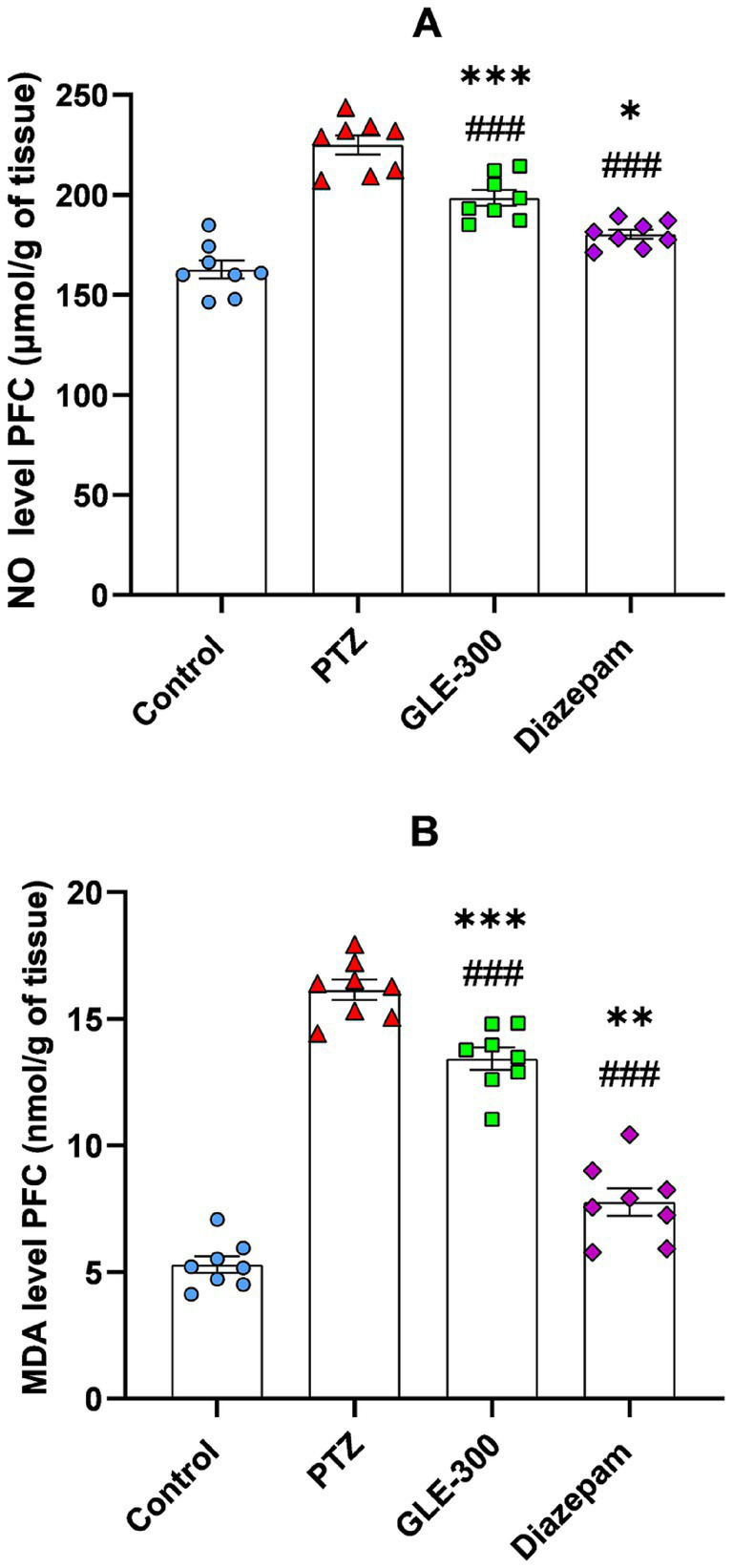
The effects of GLE and diazepam on nitric oxide (μmol/g of tissue) **(A)** and malondialdehyde (nmol/g of tissue) levels **(B)** in the prefrontal cortex. One-way ANOVA and Tukey’s post hoc test were used for the statistical analysis, and all data are shown as mean ± S.E.M (*n* = 8). *p < 0.05, **p < 0.01, and ***p < 0.001 indicates significant differences between the diazepam and GLE-300 groups and the control, elsewhere ^###^*p* < 0.001 indicates significant differences between the diazepam and GLE-300 groups and the PTZ group.

Our results show a significant variation between the experimental groups [*F* (3, 28) = 44.6639; *p* < 0.001; Figure 7A]. NO levels increased significantly in the PTZ group (225.00 ± 4.78 μmol/g of tissue), compared with the control group (162.67 ± 4.50 μmol/g of tissue), indicating a powerful state of oxidative stress (*p* < 0,001). Whereas, pretreatment of animals with GLE followed by PTZ administration significantly reduced NO levels (198.47 ± 3.57 μmol/g of tissue) compared to the PTZ group (*p* < 0.001). The GLE-300 group’s partial antioxidant activity was evident, nevertheless, as it continued to be considerably greater than the control group (*p* < 0.001).

In addition, diazepam significantly modulated NO levels (180.35 ± 2.13 μmol/g of tissue), showing a lower difference compared to the control group (*p* = 0.020), but revealing a highly significant decrease compared to the PTZ group (*p* < 0.001).

#### MDA levels

ANOVA analysis revealed significant differences between groups [*F* (3, 28) = 130.537; *p* < 0.001; Figure 7B], highlighting the impact of treatments on modulating oxidative stress. Our results show a significant increase in MDA levels in the PFC of rats treated only with PTZ (16.15 ± 0.41 nmol/g of tissue) compared to the control group (5.29 ± 0.30 nmol/g of tissue), suggesting a significant increase in PTZ-induced lipid peroxidation (*p* < 0.001). GLE significantly decreased MDA levels (13.43 ± 0.44 nmol/g of tissue), compared to the PTZ group (*p* < 0.001), implying a partial antioxidant effect of this extract. However, MDA levels in the GLE-300 group remained significantly higher than in the control group (*p* < 0.001), indicating a partial reduction in lipid peroxidation.

On the other hand, diazepam administration decreased MDA levels (7.64 ± 0.54 nmol/g of tissue), with levels significantly lower than those of the PTZ group (*p* < 0.001) and significantly higher than those of the control group (*p* = 0.002). This indicates a greater neuroprotective efficacy of GLE, while showing moderate persistence of oxidative stress.

### Catalase activity

**Figure 8:**
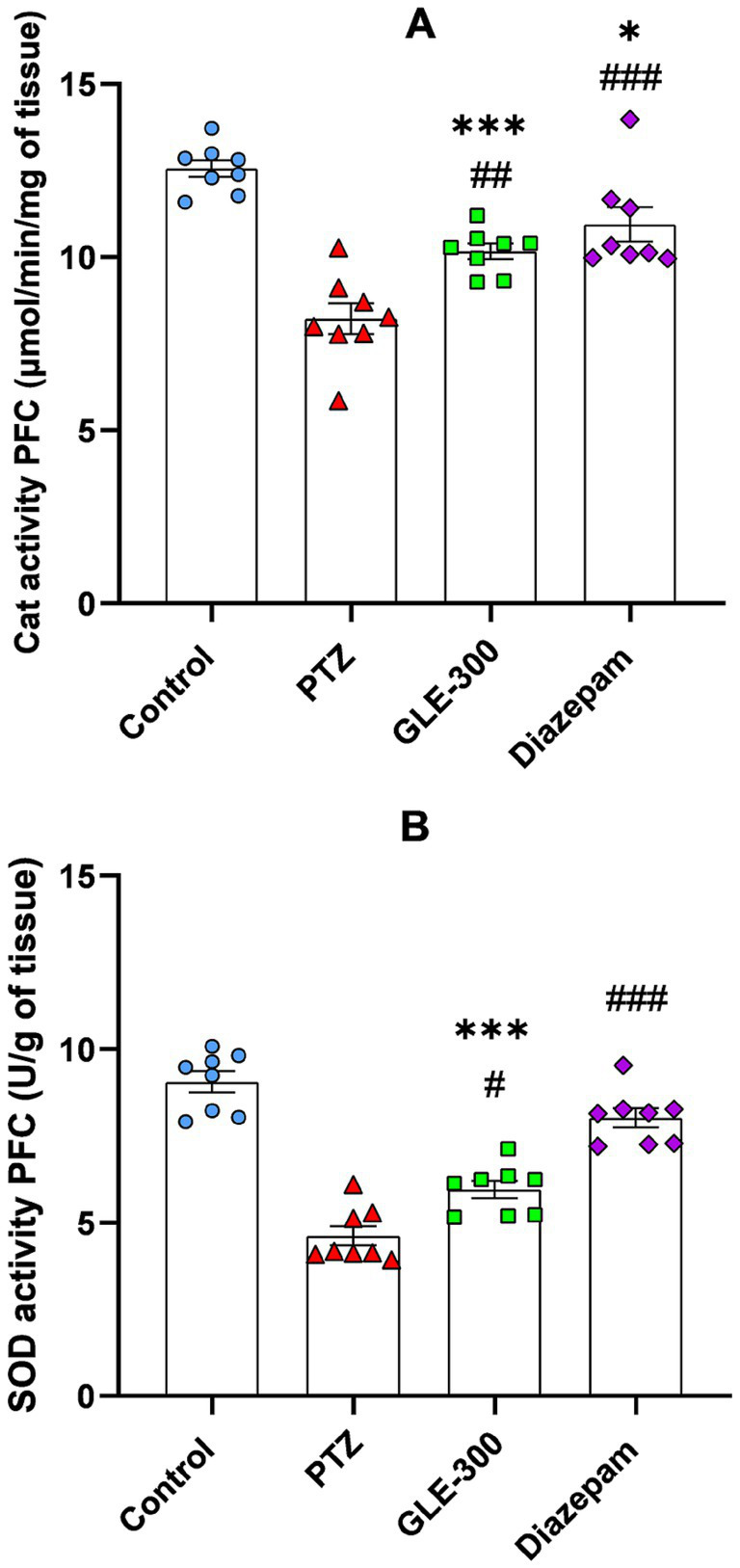
The effects of GLE on catalase (μmol/min/mg of tissue; **A**) and superoxide dismutase activity (U/g of tissue; **B**) in the prefrontal cortex. One-way ANOVA and Tukey’s post hoc test were used for the statistical analysis, and all data are shown as mean ± S.E.M (*n* = 8). *p < 0.05 and ***p < 0.001 between the diazepam and GLE-300 groups and the control, # *p* < 0.05, ## *p* < 0.01, and ###*p* < 0.001 between the diazepam and GLE-300 groups and the PTZ group.

ANOVA statistical analysis validated the presence of significant disparities between groups [*F* (3, 28) = 23.3751; *p* < 0.001; Figure 8A]. Our results indicate a considerable reduction in catalase catalytic activity in the PTZ group (8.23 ± 0.45 μmol/min/mg of tissue) compared with the control group (12.56 ± 0.24 μmol/min/mg of tissue), reflecting a major impairment of innate antioxidant mechanisms (*p* < 0.001). This enzymatic deficit is a crucial indicator of the redox imbalance resulting from PTZ-induced crises. However, animals in the GLE-300 group showed a partial increase in catalase activity (10.17 ± 0.23 μmol/min/mg tissue) compared to the PTZ group (*p* = 0.005), suggesting a moderate antioxidant effect.

Notwithstanding, diazepam promoted a significant restoration of catalase activity (10.95 ± 0.50 μmol/min/mg of tissue), with a significant change compared to the PTZ group (*p* < 0.001), and a less significant difference compared to the control group (*p* = 0.024).

### SOD activity

Our results show highly significant disparities between groups [*F* (3, 28) = 51.7648; *p* < 0.001; Figure 8B]. In this PTZ-Kindling model, chronic PTZ administration significantly reduced SOD activity at the PFC (4.62 ± 0.28 U/g of tissue) compared with the control group (9.06 ± 0.31 U/g of tissue), confirming a major seizure-induced pro-oxidant effect (*p* < 0.001).

On the other hand, in the GLE-300 group, GLE partially recovered this activity (5.96 ± 0.25 U/g of tissue), with a notable improvement over the PTZ group (*p* = 0.010), although the enzyme level remains considerably below that of the control group (*p* < 0.001). This indicates partial modulation of redox balance by GLE.

However, Diazepam restored SOD catalytic activity (8.02 ± 0.28 U/g tissue), reflecting a significant difference compared to the PTZ group (*p* < 0.001), as well as values comparable to those of the control group.

### Histological evaluations

**Figure 9:**
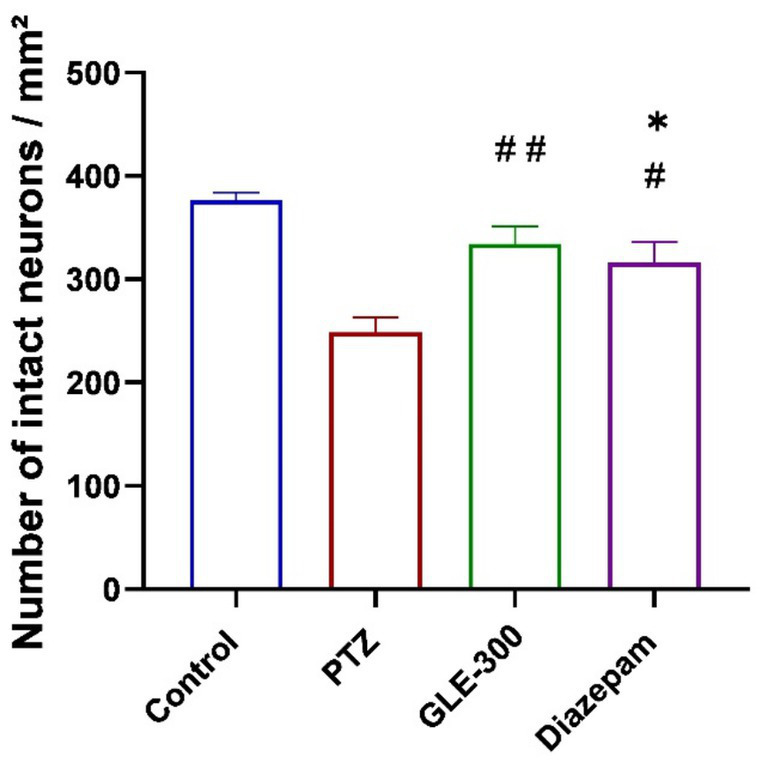
The potential neuroprotective effects of GLE and diazepam on nerve cell number at the prefrontal cortex in the PTZ-kindling model. One-way ANOVA and Tukey’s post hoc test were used for the statistical analysis, and all data are shown as mean ± S.E.M (*n* = 8). *p < 0.05 represent statistically significant differences within the diazepam and GLE-300 groups and the control group; furthermore, #*p* < 0.05 and ##*p* < 0.01 show statistically significant differences between the diazepam and GLE-300 groups and the PTZ group.

In the present study, there was a significant decrease in the number of nerve cells in the PFC of the PTZ group compared with the control group (F (3, 28) = 11.8259; *p* < 0.001; Figure 9).

In addition, the GLE-300 (*p* = 0.003) and Diazepam (*p* = 0.023) groups showed an increase in cell number compared with the PTZ group. These results are consistent with the findings of the behavioral studies.

Histopathological studies of PFC pyramidal neurons from the control groups revealed a normal histological appearance, as seen in Figure 9. In contrast, the PTZ group clearly displayed neuronal loss, cellular disarray, and cells with dense, black cytoplasm and dark, pyknotic nuclei.

**Figure 10:**
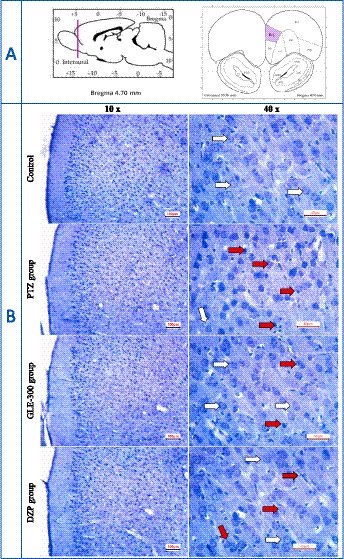
Histopathological evaluation of neurodegenerative changes in the prefrontal cortex. **(A)** Region of interest and its anatomical positions: prelimbic cortex (PrL) indicated by a dotted line. **(B)** Representative images of Nissl-stained forebrain sections 4.70 mm relative to bregma; left hemisphere; PrL of Control (PBS), PTZ (35 mg/kg; *i.p*), GLE-300 (300 mg/kg; *i.p* of GLE + 35 mg/kg of PTZ; *i.g*) and Diazepam group (5 mg/kg; *i.p* of Diazepam+ 35 mg/kg; *i.p* of PTZ). The white arrow indicates viable and intact cells characterized by clear and visible nuclei and nucleoli with a visible cytoplasm outline, while the red arrow represents degenerated cells or cells undergoing degeneration, which had dark and pyknotic nuclei with dense and dark cytoplasm (magnifications ×10 and ×40). Drawings are adapted from Paxinos and Watson (103). All coordinates for the studied subregions were obtained from the rat brain atlas (103).

When compared to the PTZ group, these alterations were less pronounced in the PTZ + GLE group, revealing normal pyramidal neurons (Figures 9, 10).

## Discussion

Our results showed that after the kindling process, the male epileptic rats, compared to the control groups, exhibited recurrent convulsive seizures and displayed anxious and depressive-like behaviors in various behavioral tests. They also showed a significant increase in NO and MDA levels in the PFC brain regions, along with a decrease in SOD and catalase enzymatic activity, with an evident association between neuronal death and abnormal behavioral performance.

The PTZ-induced kindling model is an established experimental model of chronic generalized epilepsy, widely used because of its reliability, reproducibility, and relevance in replicating certain aspects of human epilepsy (53).

Repeated administration of subconvulsive doses of PTZ induces a gradual and persistent increase in seizure susceptibility, leading to the development of severe seizures and the induction of a state of sustained neuronal hyperexcitability (54, 55). This process involves alterations in the GABAergic, glutamatergic, and antioxidant systems, leading to cellular and molecular changes associated with increased oxidative stress. In this context, the present study highlighted the neuroprotective effects of GLE on the progression of the kindling process to stage 4–5 seizures. Beyond convulsive manifestations, the progression of kindling is accompanied by functional changes within the cortical and limbic networks involved in emotional regulation, thereby promoting the emergence of affective comorbidities, particularly anxiety and depressive behaviors.

Furthermore, treatment with GLE reduced brain damage caused by the kindling process and provided significant neuroprotection against epileptogenesis induced by chronic administration of PTZ when the animals were pretreated with GLE. Administration of GLE (300 mg/kg) delayed seizure progression until the ninth PTZ injection. In the present study, the anticonvulsant potential of GLE is remarkable, as the beneficial results of GLE *in vitro* studies have also been reported (56). Furthermore, based on our previous work, we revealed via the dose–response relationship that the ideal dose for reducing seizure intensity in the PTZ-induced acute seizure animal model would correspond to 300 mg/kg (27).

In this respect, GLE’s soluble polysaccharides have neuroprotective potential against epilepsy, thanks in part to their ability to inhibit neurotoxic events in the hippocampus and cortex *in vivo* (57). On top of that, results indicate that *G. lucidum* spore powder has clinical potential as a drug to protect the brain from epilepsy (58).

Experimental studies have demonstrated the benefits of *G. lucidum* for neuronal hyperexcitability disorders such as epileptic seizures. An impressive study by Wang et al. indicates that the administration of spores helps to reduce glutamate while increasing GABA in brain structures such as the cortex and hippocampus, thereby restoring the balance of excitatory and inhibitory neurotransmission, which is essential in epilepsy (59, 60). In this context, GLE contains triterpenoids, such as ganoderic acids, known for their neuroprotective properties. Although the direct effect of ganoderic acid A on GABA and glutamate levels has not been measured, research has shown that it has an antiepileptic effect via activation of the calcium detection receptor and inhibition of pro-apoptotic pathways, positively influencing glutamatergic and GABAergic neurotransmission (19). Therefore, recent reviews indicate that the bioactive compounds in *G. lucidum*, particularly triterpenoids and polysaccharides, possess antioxidant, anti-inflammatory, and neuroprotective effects. These properties may reduce glutamatergic excitotoxicity and promote synaptic inhibition. Although further research is needed on each compound, current data support the investigation of GLE as a potential modulator of GABA/glutamate balance to prevent epileptic seizures (18).

Oxidative stress is associated with the pathophysiological mechanisms of epilepsy (61), ion channel dysfunction (62), glial dysfunction (63), immune and inflammatory factors (64) and molecular genetic mechanism (65). Besides, Anxiety is one of the many psychomotor symptoms that epileptic patients may exhibit (66).

Around 45% of epilepsy patients suffer from anxiety, which severely affects their quality of life (67). We therefore assessed the comorbidities associated with anxiety-type epilepsy in animals immediately after induction of brain damage by the kindling process. In fact, rats in the PTZ group were found to be less exploratory and more anxious in the OFT and EPM compared with the control group. Therefore, this anxious behavior was mitigated by chronic supplementation of the animals with GLE, resulting in a significant anxiolytic effect, with the animals experiencing no distress when faced with the open, illuminated arena, compared to the PTZ group.

Our results are in line with previous studies in which the action of GLE (68) as an anxiolytics has been reported by tests in various rodent behavioral models. Our results are consistent with a preclinical study that reported the potential anxiolytic effect of GLE in rats exposed to streptozotocin (69). Accordingly, these results suggest that GLE could be useful for the treatment of epilepsy-related comorbidities, including depression and anxiety, in the PTZ-kindling model of epilepsy.

Prior research has demonstrated that *G. lucidum* triterpenoids reduce oxidative stress in the brain and peripheral tissues, hence reducing anxiety and sadness in mice brought on by mother separation (70). In the chronic social defeat stress model of depression, injections of *G. lucidum* polysaccharides had a strong and quick antidepressant effect by raising brain-derived neurotrophic factor (BDNF) and inhibiting pro-inflammatory cytokine levels (71). These findings suggest that GLE possesses neuroprotective and antioxidant properties that might be linked to its antidepressant effects. Therefore, we investigated the pharmacological mechanism of action of GLE in PTZ rats from the perspectives of neuroplasticity and oxidative stress.

In our research, GLE-pretreated animals proved less depressive than rats in the PTZ group in the FST. In a previous study, Matsuzaki et al. (72) observed that GLE acted on the depressive state with diazepam in the FST. Previous studies have shown that downtime increases after the PTZ ignition process (73), License *et al* proved that supplementing rats with GLE increased the animals’ mobility in the FST, validating the antidepressant profile of this extract (74).

For this reason, the neuroprotective effects of GLE are known to act by regulating the activity of the glutamatergic and GABAergic systems, protecting against excitotoxicity-induced cell damage and the resulting neurological disorders, including epilepsy (75).

All in all, the ignition process is associated with increased MDA and NO levels in the PFC (76, 77). Recent studies on the neurotherapeutic effects of *G. lucidum* have shown that supplementation of rats with GLE not only reduced neuroexcitability (78), but also suppressed the flashover-induced increase in MDA and NO levels (79). The prefrontal cortex and hippocampus are sensitive brain regions in epilepsy, undergoing neuronal hyperexcitability due to an imbalance between glutamatergic transmission and GABAergic control (80, 81). Repeated seizures cause neuronal damage, leading to structural and functional changes that affect synaptic plasticity and the neural networks involved in cognition and memory.

Biochemical tests in our work revealed lower levels of SOD and catalase in the brains of PTZ-attacked rats, supporting the idea that PTZ-induced neuronal excitation raises oxidative stress (82, 83).

One of the primary pathogenic mechanisms causing the death of neurons in the epileptic brain is the imbalance between excitation and inhibition, which results in hyperexcitability (84). Exaggerated neuroexcitability causes high oxidative stress, which contributes to the development of epilepsy (85).

However, rats given chronic GLE attenuated this oxidative stress, and the results are consistent with previous studies that have authenticated the antioxidant potential of GLE by *in vitro* tests on brain homogenates (85, 86).

The study by Wang et al. (56) shows that replacing the nutrient environment with a magnesium-free extracellular matrix for 3 h significantly increases calcium in the cytoplasm of neurons. However, addition of GLE to the magnesium-containing extracellular matrix reduces calcium fluorescence intensity in hippocampal neurons, suggesting that GLE may inhibit calcium accumulation and increase expression of Calmodulin kinase II (CaMKIIα) (56).

The results showed that after GLE treatment, the number of normal PFC neurons increased and morphology was well preserved. In addition, catalase activity and SOD increased significantly, while MDA and NO levels decreased in the GLE-300 group compared with the PTZ group, suggesting that GLE protected cortical neurons in the prelimbic cortex by promoting antioxidant enzyme activity and inhibiting reactive oxygen species (ROS) production. This is in line with Wang’s studies proving the neuroprotective effects of GLE (87), because of the buildup of ROS and nitrogen, oxidative stress causes the death of neurons (88). Oxidative or nitrosative damage to neurons results from the brain’s vulnerability to oxidative stress, which is caused by an imbalance between antioxidant defenses and ROS generation (89). The production of NO by active microglia contributes to neuroinflammation by triggering glutamate release and encouraging N-méthyl-D-aspartate (NMDA) receptor activation, which ultimately results in neuronal death (90, 91).

The medial PFC, particularly the prelimbic cortex, is essential for regulating neuronal excitability, affective behaviors, and the spread of seizures (92). Interconnected with limbic structures such as the hippocampus and amygdala, it plays a key role in epileptogenesis and affective comorbidities associated with epilepsy (93). The pyramidal neurons of the prefrontal cortex, rich in glutamatergic receptors, are vulnerable to excitotoxicity caused by repeated seizures resulting from an imbalance between the excitatory and inhibitory systems (94).

Structural and functional alterations in the PFC have been observed in various experimental models of epilepsy, contributing to the generalization of seizures and to anxiety and depressive disorders in epileptic patients (95). The study of this region is therefore crucial to understanding the neural mechanisms linking epileptic hyperexcitability and affective dysfunction.

Although diazepam effectively suppressed seizures, its prolonged GABAergic action may induce decreased neural activity and activity-dependent plasticity adaptations (96). Studies have shown that repeated administration of benzodiazepines can cause subtle morphological changes, such as a reduction in dendritic or synaptic density (97). This results in an apparent decrease in the number of intact cells during histological analyses, regardless of convulsive activity (98). On the other hand, their ability to prevent neuronal death in the prefrontal cortex is limited. They act symptomatically by potentiating GABAergic inhibition, without targeting the mechanisms of neurotoxicity associated with repeated seizures, such as glutamatergic excitotoxicity and oxidative stress (99, 100). Repeated administration of benzodiazepines can lead to pharmacological tolerance, reducing their long-term effectiveness without preventing the cumulative neuronal changes observed in kindling models (101, 102).

Although GLE supplementation reduced the severity of seizures in the PTZ kindling model, it did not completely prevent generalized seizures. GLE attenuates seizure-related anxiety and depressive behaviors, without fully normalizing parameters relative to controls. The relationship between generalized seizures and affective comorbidities has not been directly examined, suggesting that future research is needed to clarify the impact of different seizure types on anxiety and depression, as well as the neurobiological mechanisms involved.

Our results indicate that GLE could be a promising treatment for preventing and managing depression associated with epilepsy. Since epilepsy is commonly linked to psychiatric disorders such as depression, it increases overall morbidity in patients. GLE’s bioactive compounds, such as triterpenes, polysaccharides and antioxidants, could have a neuroprotective and mood-regulating role. This is due to its ability to modulate oxidative stress and excitotoxicity related to emotional circuits. Pretreatment with GLE modulates the neurochemical and behavioral changes induced in the PTZ-kindling model, thereby reducing the associated depressive symptoms.

## Conclusion

The present study concludes that during the kindling process, neurobehavioral and neurochemical changes, as well as histopathological alterations, were observed in the prelimbic cortex of the PFC. However, the results of this study suggest that GLE exerts anticonvulsant, antioxidant, and neuroprotective effects in the PTZ-induced kindling model, thereby contributing to the attenuation of anxiety-depressive alterations. These effects appear to be associated with a reduction in oxidative damage in the PFC. Thus, GLE may contribute to the preservation of redox balance and neuronal function in cortical regions involved in emotional regulation.

## Data Availability

The original contributions presented in the study are included in the article/supplementary material, further inquiries can be directed to the corresponding author.

## Glossary

GLE: *Ganoderma lucidum* extract
PTZ: Pentylenetetrazol
OFT: Open field test
FST: Forced swim test
EPM: Elevated plus test
MDA: Malondialdehyde
NO: Nitric oxide
SOD: Superoxide dismutase
NBT: Nitro blue tetrazolium
TBA: Thiobarbituric acid
PFC: Prefrontal cortex
GABA: *γ*-aminobutyric acid
*G. lucidum*: *Ganoderma lucidum*
PBS: Phosphate buffered saline
DW: Distilled water
i.g: Intragastric
i.p: Intraperitoneal
EOA: Entries in open arms
TOA: Time in open arms
NRC: Number of return to the center
TCA: Time in the central area
ROS: Reactive oxygen species
SEM: Standard error of mean
BDNF: Brain derived neurotrophic factor
NMDA: N-méthyl-D-aspartate
CaMK II: Calmodulin kinase II
